# Panoramic Assessment of Root Development in Immature Maxillary Incisors After Treatment with Prefabricated Functional Appliances

**DOI:** 10.3390/children12101416

**Published:** 2025-10-20

**Authors:** Wonbin Seo, Soyoung Park, Eungyung Lee, Taesung Jeong, Jonghyun Shin

**Affiliations:** 1 Department of Pediatric Dentistry, Dental Research Institute, Pusan National University Dental Hospital, Yangsan 50612, Republic of Korea; seobeean@naver.com (W.S.); syparkpedo@pusan.ac.kr (S.P.); eungyung@pusan.ac.kr (E.L.); tsjeong@pusan.ac.kr (T.J.); 2 Department of Pediatric Dentistry, School of Dentistry, Dental and Life Science Institute, Pusan National University, Yangsan 50612, Republic of Korea

**Keywords:** early orthodontic treatment, maxillary incisor, prefabricated functional appliance, root development

## Abstract

**Highlights:**

**What are the main findings?**

- Final root length and root-to-crown ratio showed no significant differences between the PFA-treated and control groups.
- Neither initial root stage (Nolla 8 or 9) nor appliance wear duration influenced the maturation of immature roots.

**What is the implication of the main finding?**

- Early PFA treatment appears safe for mixed-dentition children, as it does not affect the normal development of immature roots.
- PFAs may be suitable for correcting skeletal Class II and functional anterior crossbite when root growth is regularly monitored.

**Abstract:**

Background: Prefabricated functional appliances (PFAs) are widely used for interceptive orthodontic treatment in children, delivering intermittent forces with potential advantages for oral function. This study evaluated the effects of PFA treatment on the root development of maxillary central incisors in children during the mixed dentition stage using panoramic radiographs. Methods: A retrospective review was conducted on 77 children in the mixed dentition phase (2020–2025). These were divided into three groups: untreated controls (*n* = 33); skeletal Class II malocclusion group, treated with the Pre-Ortho^®^ Type 1 appliance (Group 1, *n* = 25); and functional anterior crossbite group treated with a Type 3 appliance (Group 2, *n* = 19). All participants underwent at least two panoramic radiographs; treatment was initiated when the maxillary central incisors were at Nolla stages 8 or 9. Following root completion, panoramic images were analyzed using the modified Lind method to measure crown length, root length, and root-to-crown ratio. Results: Mean final root lengths of the maxillary central incisors were 14.11 ± 1.40 mm (controls), 14.46 ± 1.42 mm (Group 1), and 13.89 ± 1.04 mm (Group 2) (*p* > 0.05). The mean root–crown ratios showed no significant variation (*p* > 0.05). Root development was unaffected by wear duration, with limited sex differences. Conclusions: PFA treatment may not adversely affect the root development of maxillary central incisors and indicates a safe intervention in children.

## 1. Introduction

Recent advancements have intensified interest in the efficacy and clinical necessity of early orthodontic treatment. Specifically, the timing of treatment plays a crucial role in the overall success of orthodontic outcomes. Appropriately timed early treatment effectively addresses both functional impairments and esthetic concerns [1].

Modulating craniofacial growth during development prevents complex and invasive procedures in adulthood. This represents a major advantage of early orthodontic intervention. In pediatric orthodontics, therapeutic modalities have diversified to include functional appliances, removable appliances, and clear aligners, in addition to conventional fixed appliances. Functional appliances are highly effective for correcting skeletal malocclusions during the prepubertal growth phase. Their success depends on patient compliance, as they facilitate the regulation of jaw growth patterns [2].

Prefabricated functional appliances (PFAs) can improve soft tissue function, optimize tongue posture, and increase upper airway volume, thereby addressing etiological contributors to malocclusion. Clinically, PFAs offer substantial advantages by eliminating the need for dental impressions or laboratory fabrication [3]. Removable appliances exert intermittent orthodontic forces, which are associated with a lower risk of orthodontically induced external apical root resorption (OIEARR) compared with continuous force application [4]. PFAs similarly deliver intermittent forces, distinguishing them from fixed appliances. Although the risk of OIEARR under continuous orthodontic forces is well-established, the impact of the intermittent forces delivered by PFAs on developing roots remains unclear. These appliances deliver physiologic, low-intensity forces by virtue of their structural design. PFAs equipped with vestibular and lingual flanges effectively mitigate excessive pressure from perioral muscles. In addition, the integrated tongue-up plate guides the tongue to an optimal anterior palatal position, thereby promoting the reestablishment of correct swallowing patterns [5]. PFAs are effective in reducing overjet and improving the A point–Nasion–B point (ANB) angle in patients with skeletal Class Ⅱ malocclusion [3] and have also proven effective in correcting functional anterior crossbite [5].

OIEARR is a common complication of orthodontic therapy, characterized by radiographic root shortening or morphological changes [6]. Maxillary central incisors exhibit heightened susceptibility to root resorption due to their anatomical configuration and the magnitude of orthodontic force typically applied [7,8]. These teeth are critical to both facial esthetics and occlusal function.

Root development begins after crown formation and continues for approximately 2–3 years post-eruption, involving a complex interplay of biological processes. Developing roots are highly susceptible to mechanical or orthodontic forces, which may lead to root shortening or deformation [9]. Concerns persist regarding the application of orthodontic forces to immature roots, as this may increase the risk of resorption or induce premature apical closure [10]. Additionally, anatomical factors, such as malocclusion type or proximity of root apices to cortical bone, also influence root development. For instance, in Class Ⅲ malocclusion or Class II division 2 cases, roots may approximate the cortical bone or teeth may be inclined palatally, thus limiting the available space for root elongation and exacerbating root resorption [7,11,12]. Consequently, periodic acquisition of progress radiographs during treatment is recommended to monitor root development.

Multiple radiographic modalities are used for diagnosing root resorption, among which cone-beam computed tomography (CBCT) offers high diagnostic accuracy [13]. However, due to increased radiation sensitivity in pediatric patients, panoramic radiography remains the preferred imaging technique in accordance with the as low as reasonably achievable (ALARA) principle. This modality enables comprehensive visualization of the dentition in a single exposure [14]. Panoramic radiographs demonstrate consistent vertical magnification (approximately 5%) and high reproducibility of linear measurements, rendering them suitable for the long-term evaluation of root development [15].

Radiographic investigations evaluating the impact of PFAs on developing roots remain extremely limited. The biomechanical force delivery of PFAs differs fundamentally from that of fixed appliances or conventional functional appliances requiring extended wear durations [16]. PFAs are typically worn for about 1 h during the day and overnight [17]. This schedule provides sufficient rest periods without orthodontic force application, allowing for physiological recovery of the periodontal ligament and surrounding tissues [6]. However, the impact of such intermittent loading on the maturation of immature roots remains inadequately elucidated.

Accordingly, this study aimed to evaluate the effects of early orthodontic treatment using PFAs on the root development of maxillary central incisors in children during the mixed dentition phase, based on panoramic radiographic analysis. Furthermore, through conducting comparisons with an untreated control group, this investigation assesses the safety profile of PFA therapy and supports the expansion of clinical indications for early orthodontic management.

## 2. Materials and Methods

### 2.1. Study Design and Participants

In this retrospective study, we included pediatric patients treated between May 2020 and May 2025. Eligibility required the availability of at least two high-resolution panoramic radiographs—either pre-treatment or taken during the observational interval—acquired using standardized imaging protocols.

Three groups were defined for comparative analysis: an untreated control group and two intervention groups treated with interceptive orthodontic treatment using Pre-Ortho^®^ Type 1 or Type 3 PFAs (Pre-Ortho^®^; OptimaOrtho Korea, Pusan, Republic of Korea). Patient data were extracted from electronic medical records following the application of defined inclusion and exclusion criteria.

The inclusion criteria were as follows: children in the mixed dentition phase; children with panoramic radiographs of diagnostically acceptable quality; those without prior history of orthodontic treatment; and those with maxillary central incisors exhibiting complete root formation and closed apical foramina (Nolla stage 10), confirmed via panoramic radiographs at the time of root length measurement.

The exclusion criteria were as follows: children with systemic disorders affecting the oral and maxillofacial region; those with inadequate patient compliance including missed appointments or failure to follow appliance instructions; those with a history of dentoalveolar trauma; those with a history of previous endodontic therapy or restorative procedures involving the maxillary central incisors; and those that had undergone surgical intervention in the anterior maxillary region.

### 2.2. Control Group

The control group comprised pediatric patients who underwent routine panoramic radiographic examinations at the Department of Pediatric Dentistry during the same observational period as that of the treatment groups. Records were meticulously screened, and only patients with comparable chronological age at the conclusion of the observation period were included to minimize potential bias in root development. Ultimately, 33 patients (15 males, 18 females; mean age: 11.38 ± 0.91 years) were enrolled in the control group.

### 2.3. Treatment Group

To ensure sample homogeneity by excluding patients with differing root development trajectories, patients whose maxillary central incisors were at Nolla stage < 8 (i.e., less than two-thirds root formation) or stage 10 (complete apical closure) at baseline were excluded.

**Figure 1:**
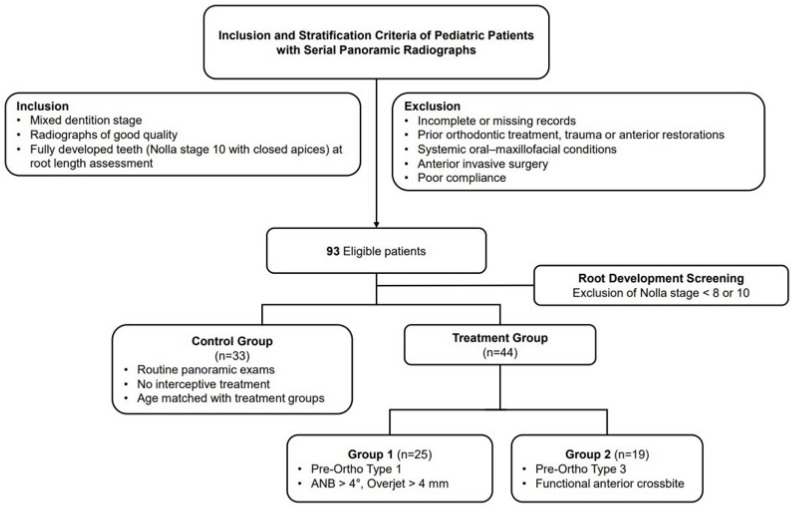
Flow diagram of patient selection and grouping in this study.

Group 1 (Type 1 PFA), characterized by skeletal Class II malocclusion, included 25 patients (13 males, 12 females; mean age 11.03 ± 0.78 years) who met the following diagnostic criteria: (i) pretreatment overjet of >4 mm, and (ii) ANB angle > 4°. Group 2 (Type 3 PFA), characterized by functional anterior crossbite, included 19 patients (10 males, 9 females; mean age 10.98 ± 0.76 years), who met the following diagnostic criteria: (i) presence of one or more maxillary incisors in crossbite and (ii) demonstrating an edge-to-edge incisal relationship upon guided mandibular closure. The recruitment and selection process of the study participants is illustrated in Figure 1, detailing the inclusion and exclusion criteria applied to each treatment group. Ultimately, participants were stratified into three distinct cohorts: Control (*n* = 33), Group 1 (*n* = 25), and Group 2 (*n* = 19). Particular emphasis was placed on achieving homogeneity in chronological age, sex distribution, and root developmental stage to minimize confounding variables.

The prescribed wear protocol for the Pre-Ortho^®^ appliance involved 1 h of daytime use and continuous wear overnight. Appliance size (S or M) was determined via evaluation of dental casts in accordance with the manufacturer’s guidelines. To improve compliance and reduce initial discomfort, the soft-type appliance was preferentially prescribed at the beginning of treatment. Parents or guardians were instructed to supervise daily wear and reinforce adherence to the regimen.

### 2.4. Study Methods

A total of 77 panoramic radiographs (of 38 male and 39 female children) were analyzed, encompassing 154 maxillary central incisors that had attained complete root formation with apical closure (Nolla stage 10). To minimize overlap errors, all panoramic radiographs were acquired using a single imaging unit (Proline XC; Planmeca Co., Helsinki, Finland). Digitized panoramic radiographs were imported into the image analysis software (ImageJ version 1.54; Bethesda, MD, USA). Calibration was performed using the Set Scale function, and all measurements were recorded in millimeters (mm).

**Figure 2:**
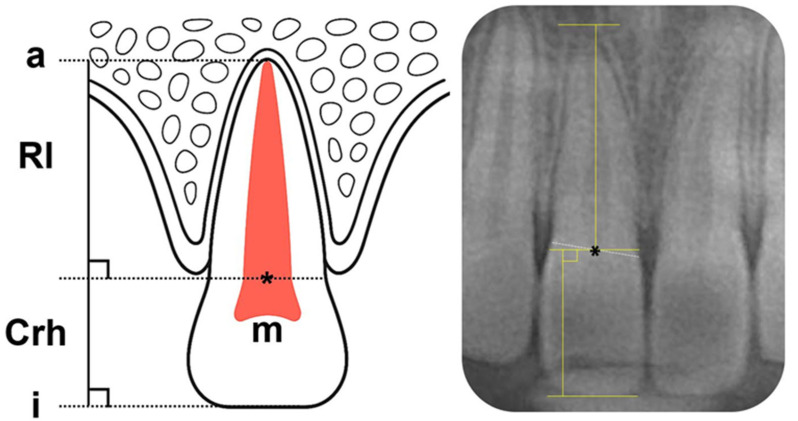
Modified Lind method for the measurement of crown height and root length. i: incisal level; a: apical level; Rl: root length; Crh: crown height; m: midpoint of a straight line that connects the points of intersection between the outer contours of the root and crown. Asterisk (*) indicates the midpoint (m).

To accommodate the limitations of panoramic imaging, specifically the indistinct visualization of the cemento-enamel junction (CEJ), teeth were measured using a modified version of Lind’s measurements for the precise measurement of crown height and root length [18]. Based on this technique, the midpoint (point M) of a straight line connecting the points of intersection between the outer contours of the crown and root was designated as the reference point. An occlusal reference line was drawn along the incisal edge. Crown height was defined as the perpendicular distance from point M to the incisal reference line, and root length was defined as the perpendicular distance from point M to the apical reference line [19] (Figure 2).

The root-to-crown ratio (R/C ratio) may be defined anatomically using the CEJ or clinically using the alveolar crest level. Given the variability of alveolar crest position due to growth, remodeling, or resorption, the clinical definition may not accurately reflect the true R/C ratio of teeth undergoing development or functional activity. Therefore, this study adopted the anatomical CEJ-based definition to eliminate the influence of individual variation in periodontal condition on the experimental data, consistent with previous studies [12].

**Figure 3:**
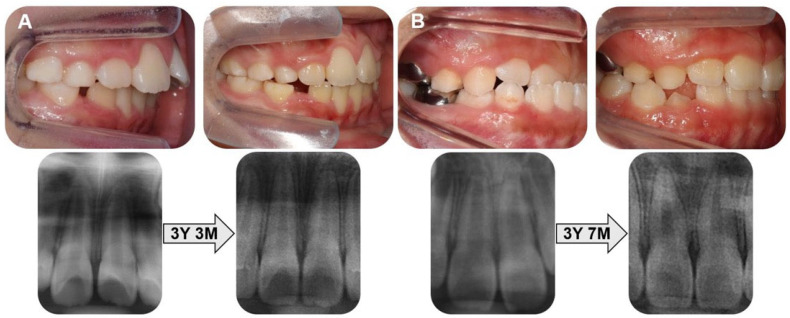
Pre- and post-treatment intraoral photographs and panoramic radiographs. (**A**) Patient with Class II malocclusion. (**B**) Patient with functional anterior crossbite.

Representative pre- and post-treatment intraoral photographs and panoramic radiographs are presented in Figure 3.

### 2.5. Statistical Analysis

Normality of data distribution was assessed using the Shapiro–Wilk test. Data are presented as the mean ± standard deviation (SD). To eliminate inter-examiner variability, all radiographic measurements were performed by a single, calibrated examiner (W.S.) who was blinded to group allocation. The reproducibility of this examiner was evaluated by re-measuring 30 randomly selected radiographs after a two-week interval, and intra-examiner reliability was quantified using intraclass correlation coefficients (ICCs). Intergroup comparisons (control, Group 1, Group 2) were conducted using one-way analysis of variance (ANOVA) or the Kruskal–Wallis test, contingent upon data normality. Differences related to sex and Nolla stage were analyzed using the independent *t*-test or Mann–Whitney U test, as appropriate. Correlations between appliance wear duration and root development parameters (mean root length and R/C ratio) were evaluated using Spearman’s rank correlation coefficient. All statistical tests were two-tailed with a significance threshold of *α* = 0.05, and analyses were performed using SPSS Statistics version 29.0 (IBM Corp., Armonk, NY, USA).

## 3. Results

Complete datasets were obtained from 77 children in the mixed dentition phase, with a mean chronological age of 11.17 ± 0.85 years. The sex distribution was nearly equal: 38 male (49.4%) and 39 female children (50.6%) (Table 1). The mean chronological age at radiographic evaluation was 11.38 ± 0.91 years in the control group, 11.03 ± 0.78 years in Group 1, and 10.98 ± 0.76 years in Group 2. The other demographic characteristics of the participants are presented in Table 1. To ensure consistency in developmental staging, only patients at Nolla stage 8 or 9 at treatment onset were included.

The ICC values for crown height, root length, and R/C ratio of the maxillary central incisors ranged from 0.93 to 0.98, demonstrating excellent intra-examiner reliability. Consistent with previous studies, bilateral measurements were averaged and used for all sub-sequent analyses.

**Figure 4:**
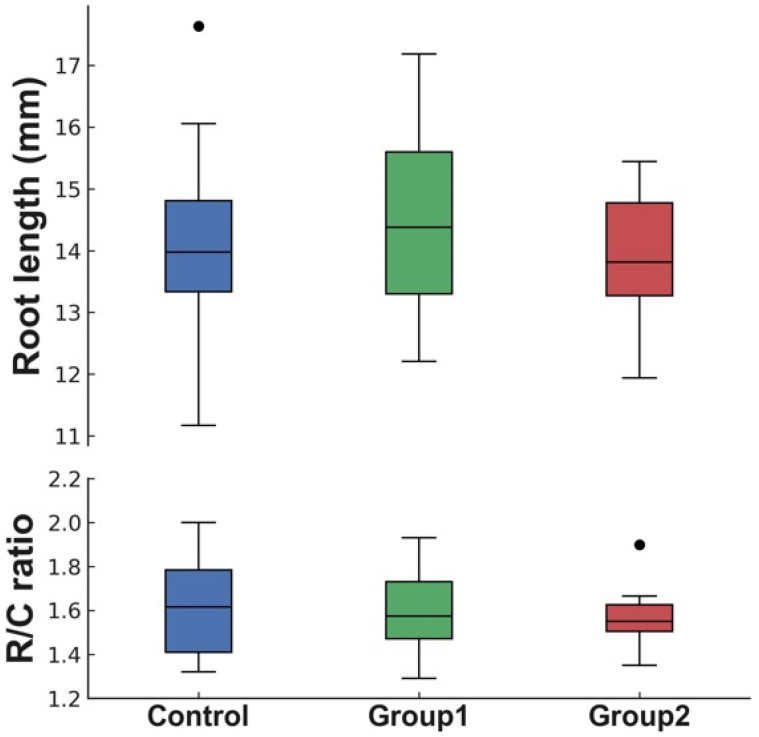
Boxplots of root length and root–crown ratio of maxillary central incisors in the control group, Group 1, and Group 2.

Groupwise comparisons of the maxillary central incisor measurements are presented in Table 2 and Figure 4. None of these differences reached statistical significance.

The calculated effect sizes were small, with Cohen’s *f* = 0.17 for root length and 0.13 for R/C ratio in the three-group comparisons, indicating minimal differences between groups.

Similarly, when comparing the two treatment groups, small-to-moderate effects were found (Cohen’s *d* = 0.45 for root length, 0.27 for R/C ratio), suggesting that these differences were not clinically significant.

The 95% confidence intervals of group means largely overlapped, further supporting the absence of meaningful intergroup variation.

No significant differences in root length or R/C ratio were observed between subjects at Nolla stage 8 and 9 in either Group 1 or Group 2 (all *p* > 0.05) (Table 3).

In Group 2, male children exhibited greater crown height than that in females for both the right (males vs. females: 9.13 ± 0.72 mm vs. 8.50 ± 0.53 mm; *p* = 0.002) and left incisors (males vs. females: 9.37 ± 0.49 mm vs. 8.62 ± 0.51 mm; *p* = 0.001), whereas the left R/C ratio was lower in males (1.47 ± 0.07) than in females (1.59 ± 0.14; *p* = 0.002). No other significant sex-related differences were identified (Table 4).

No significant correlation was found between appliance wear time and either mean root length (*ρ* = 0.11, *p* = 0.48) or R/C ratio (*ρ* = 0.19, *p* = 0.21). This indicates that the duration of appliance use did not have a meaningful impact on root development.

## 4. Discussion

This study evaluated the effects of early orthodontic treatment with PFAs on the root development of maxillary central incisors in children by using panoramic radiographs. No significant differences were found between the treatment and control groups in the final root length or R/C ratio. Neither the initial root stage nor the duration of appliance wear significantly affected root growth. These results are consistent with previous reports indicating that long-term appliance use does not increase the risk of root resorption [4,16]. However, the results should be interpreted with the consideration of potential bias due to the limitations in measuring wear time. These findings suggest that early treatment with PFA may not adversely affect the normal maturation of developing maxillary central incisor roots. PFAs facilitate malocclusion correction by enhancing perioral muscle function, stimulating mandibular growth, and maintaining upper airway patency [20]. When introduced during early mixed dentition, PFAs guide the harmonious growth of both soft and hard tissues [21]. Specifically, in skeletal Class II malocclusion, PFAs may promote mandibular growth and restrain maxillary overgrowth, thereby reducing the risk of trauma to the maxillary anterior teeth and enhancing facial esthetics [22]. Early intervention is recommended for anterior crossbite, as untreated cases may progress to skeletal discrepancies [23].

OIEARR is a common complication in orthodontic treatment, characterized by apical root shortening and morphological alterations detectable via radiographic imaging [6]. Maxillary anterior teeth are particularly vulnerable due to their anatomical configuration and the magnitude of applied orthodontic forces [7], although they play key functional and esthetic roles. Orthodontic loading compresses the periodontal ligament and reduces blood flow, thereby activating osteoclasts and resulting in resorption of the root surface cementum and dentin [24]. A 3 mm reduction in apical root length is considered biologically equivalent to a 1 mm loss in alveolar bone height [25].

No radiographic evidence of root resorption was observed, likely due to the intermittent and low-magnitude forces generated by PFAs, unlike the continuous forces of fixed appliances. Intermittent force application has been associated with a lower risk of root resorption [4], as it permits physiological recovery and cementum remodeling during force-free intervals [6]. Similar trends have been reported with other removable functional appliances such as clear aligners, activators, and twin-blocks [26].

To mitigate selection bias inherent to retrospective study designs, stringent inclusion criteria and balanced demographics were applied. Furthermore, acknowledging that the stage of root development may influence treatment outcomes [27,28], we included only patients at Nolla stages 8 or 9 to enhance intergroup comparability.

The mean age at panoramic imaging was 11.17 ± 0.85 years, aligning with the average developmental age of Korean children [29] and corresponding to the stage when root formation approaches completion [30]. Accordingly, the maxillary central incisors in this study were near the completion of root development, supporting the validity of intergroup comparisons. These findings suggest that PFA therapy may be safely applied even in teeth approaching the completion of root formation. However, given prior reports indicating an increased risk of root resorption when orthodontic treatment commences after age 11 [31], long-term follow-up is warranted.

Panoramic radiographs were used for measurement to comply with the ALARA principle and are commonly used in clinical practice [14]. However, due to the inherent limitations of two-dimensional imaging, such as magnification and distortion [32], the modified Lind’s method [12,33] was employed to address challenges related to indistinct visualization of the cemento-enamel junction and variability in alveolar crest levels. The R/C ratio was utilized as the primary analytic metric instead of absolute root length, given its reduced susceptibility to distortion and its high reproducibility and reliability in panoramic imaging [15].

The final average root lengths of the maxillary central incisors were 14.46 ± 1.42 mm in Group 1, 13.89 ± 1.04 mm in Group 2, and 14.11 ± 1.40 mm in the control group, with no significant differences between the three groups. Although Group 2 showed a slightly shorter mean root length (13.89 mm) than the control group (14.11 mm), the difference was not statistically significant. Given the small effect size (Cohen’s *f* = 0.17) and limited sample size, this minor variation is unlikely to be clinically meaningful, though the possibility of a Type II error cannot be completely ruled out. Further studies with larger samples are needed to clarify its biological or clinical relevance. The R/C ratios were 1.60 ± 0.18 in Group 1, 1.56 ± 0.12 in Group 2, and 1.62 ± 0.20 in the control group. These values were slightly higher than the average reported for young Korean adults (1.49 ± 0.20) by Yun et al. [33] but were lower than those documented for adult Finnish males (1.86) and females (1.78) [34]. These findings underscore the importance of considering racial and ethnic variations in orthodontic diagnosis and treatment planning. Additionally, they support the conclusion that approximately 2 years of PFA therapy does not appear to hinder the normal root development of immature maxillary central incisors. However, these interethnic comparisons should be interpreted with caution, as the cited studies differ in methodology, sample characteristics, and age distribution.

The initial stage of root development at treatment initiation (Nolla stage 8 or 9) did not significantly affect the final root length in either treatment group.

Root development typically continues for 2–3 years after eruption [9]. Although concerns persist regarding the potential for orthodontic forces to impair root development in immature teeth, recent studies have reported contradictory findings [35]. The presence of uncalcified predentine and Hertwig’s epithelial root sheath in immature roots may confer resistance to resorptive cellular activity [10], while the increased elasticity of surrounding alveolar bone may attenuate mechanical stress during orthodontic tooth movement. Furthermore, the rich vascularization and high cellular activity of the apical papilla may facilitate tissue regeneration and continuous elongation, even under light intermittent forces such as those generated by PFAs [36]. Some studies have even reported accelerated root growth in response to orthodontic force [37]. In this study, teeth at Nolla stage 8 or 9 exhibited final root lengths comparable to those in the control group, indicating that PFA therapy did not compromise growth potential. These results align with prior findings of root length augmentation following twin-block therapy [38] and the absence of adverse effects on incisor development during early treatment with 2 × 4 fixed appliances [9]. Although fixed appliances exert continuous forces, studies have shown that light, well-controlled forces allow immature teeth to complete root formation without adverse effects [27]. Therefore, the timing of early PFA intervention is unlikely to negatively influence final root length.

In Group 2, the observation that males exhibited greater crown height and a lower R/C ratios compared with females aligns with well-established sexual dimorphism in dental morphology, rather than being an artifact of the PFA treatment [39]. Such differences may reflect earlier dental and skeletal maturation in females during the mixed dentition stage [40].

Although previous studies have linked certain malocclusion types—such as skeletal Class Ⅲ or Class II division 2—with reduced root length or unfavorable R/C ratios [7,11], this study found no significant correlation between malocclusion severity and final root length. This lack of association may reflect the mild severity of malocclusions in our sample, which likely did not influence root growth or impose excessive mechanical stress during PFA therapy.

This study’s retrospective design is subject to inherent limitations. Although efforts were made to mitigate bias through stringent selection criteria and group balancing, confounders such as oral habits and growth patterns could not be fully controlled. Furthermore, the small sample size of the subgroups may be insufficient to detect subtle differences, thus limiting the statistical power of the analysis. Consequently, the findings should be interpreted with caution. In addition, reliance on two-dimensional panoramic radiographs without CBCT validation is a notable limitation. This approach restricts the detailed evaluation of root morphology, three-dimensional volumetric changes, and buccal or lingual surface involvement. Recent advances in artificial intelligence offer new possibilities for improving radiographic evaluation. These technologies can enhance diagnostic precision and reproducibility by helping to overcome the limitations of two-dimensional imaging through automated interpretation and pattern recognition [41]. The lack of quantified appliance-wear data may also have introduced measurement bias. Therefore, future prospective studies employing CBCT-based assessments, larger and more diverse cohorts, and objective compliance tracking are warranted to validate the long-term stability and clinical efficacy of PFA treatment.

Within these methodological constraints, our findings suggest that approximately 2 years of early orthodontic treatment using PFAs in children during the mixed dentition stage did not show any measurable negative impact on the root development of immature maxillary central incisors. While these results are encouraging, they should be interpreted with caution given the study’s retrospective design and limited sample size.

These findings suggest that PFAs may be a safe and viable short-term modality for early intervention in certain malocclusions. However, it is crucial that root maturation is routinely monitored through radiographic and clinical assessments. The present results have potential practical implications for patient counseling, risk assessment, radiographic monitoring strategies, and guidelines on the timing of interceptive treatment. Furthermore, the integration of AI-driven diagnostic tools in future research is warranted to enhance the precision and consistency of radiographic assessments.

## Figures and Tables

**Table 1:** Demographic characteristics of the study participants.

Variable	Total (*n* = 77)	Control (*n* = 33)	Group 1 (*n* = 25)	Group 2 (*n* = 19)	*p* Value
Chronological age (yr)	11.17 ± 0.85	11.38 ± 0.91	11.03 ± 0.78	10.98 ± 0.76	0.163
Sex					0.838
Male	38 (49.4%)	15 (45.5%)	13 (52.0%)	10 (52.6%)	
Female	39 (50.6%)	18 (54.5%)	12 (48.0%)	9 (47.4%)	
Initial age (yr)			8.82 ± 1.23	8.44 ± 1.07	
Wear time (yr)			2.08 ± 0.81	1.91 ± 0.74	
Nolla stage					
8	22 (50.0%)		11 (44.0%)	11 (57.9%)	
9	22 (50.0%)		14 (56.0%)	8 (42.1%)	

Chronological age: the mean age at the time of radiographic evaluation; Initial age: the mean age at the time of appliance delivery; Wear time: the mean duration of appliance wear. Data are presented as the mean ± standard deviations or as numbers (percentages). *p* values were obtained using one-way ANOVA or the Chi-square test, as appropriate.

**Table 2:** Comparison of crown height, root length, and root–crown ratio between the control and treatment groups.

Variable	Control		Group 1		Group 2		*p* Value
	Rt Lt	Mean	Rt Lt	Mean	Rt Lt	Mean	
Crown height (mm)	8.76 ± 0.63 8.80 ± 0.54	8.78 ± 0.56	9.04 ± 0.59 9.06 ± 0.47	9.05 ± 0.17	8.83 ± 0.71 9.01 ± 0.63	8.92 ± 0.12	0.087 ^†^
Root length (mm)	14.16 ± 1.46 14.07 ± 1.53	14.11 ± 1.40	14.52 ± 1.68 14.40 ± 1.26	14.46 ± 1.42	14.04 ± 1.18 13.75 ± 1.04	13.89 ± 1.04	0.201 ^†^
Root–crown ratio	1.63 ± 0.22 1.60 ± 0.21	1.62 ± 0.20	1.61 ± 0.21 1.59 ± 0.17	1.60 ± 0.18	1.59 ± 0.14 1.53 ± 0.12	1.56 ± 0.12	0.580 ^†^

Data are presented as the mean ± standard deviation. Right (Rt) and left (Lt) values were measured separately within each group, and their arithmetic mean was used for all subsequent analyses. Normality was tested using the Shapiro–Wilk test. Intergroup comparisons were performed using one-way ANOVA for normally distributed variables and the Kruskal–Wallis test for variables that violated normality. ^†^: Kruskal–Wallis test.

**Table 3:** Comparison of crown height, root length, and root–crown ratio according to Nolla stage.

Nolla		Group 1		*p* Value	Group 2		*p* Value
		Stage 8	Stage 9		Stage 8	Stage 9	
Crown height (mm)	Rt	8.95 ± 0.46	9.12 ± 0.68	0.603 ^†^	8.94 ± 0.85	8.69 ± 0.49	0.480
	Lt	9.15 ± 0.42	8.99 ± 0.51	0.400	9.05 ± 0.73	8.96 ± 0.51	0.749
	Mean	9.05 ± 0.41	9.05 ± 0.54	0.996	8.99 ± 0.56	8.82 ± 0.35	0.556
Root length (mm)	Rt	14.84 ± 1.83	14.26 ± 1.57	0.420	13.97 ± 1.39	14.12 ± 0.89	0.780
	Lt	14.64 ± 1.58	14.21 ± 0.95	0.441	13.82 ± 1.23	13.64 ± 0.78	0.702
	Mean	14.74 ± 1.21	14.23 ± 0.92	0.413	13.90 ± 0.93	13.88 ± 0.81	0.974
Root–crown ratio	Rt	1.66 ± 0.19	1.57 ± 0.22	0.327	1.57 ± 0.18	1.62 ± 0.06	0.369
	Lt	1.61 ± 0.20	1.59 ± 0.15	0.794	1.53 ± 0.15	1.53 ± 0.09	0.932
	Mean	1.63 ± 0.19	1.58 ± 0.18	0.499	1.55 ± 0.15	1.58 ± 0.06	0.621

Data are presented as the mean ± standard deviation. Comparisons between Nolla stage 8 and stage 9 within each treatment group were performed using the independent *t*-test, except for non-normally distributed variables (^†^), for which the Mann–Whitney U test was applied.

**Table 4:** Comparison of crown height, root length, and root–crown ratio according to sex.

Variable	Control		Group 1		Group 2	
	Rt	Lt	Rt	Lt	Rt	Lt
Crown height (mm)						
Male	8.91 ± 0.63	8.95 ± 0.51	9.06 ± 0.57	9.13 ± 0.51	9.13 ± 0.72	9.37 ± 0.49
Female	8.63 ± 0.62	8.68 ± 0.55	9.13 ± 0.50	8.98 ± 0.42	8.50 ± 0.53	8.62 ± 0.51
*p* value	0.218 ^†^	0.165 ^†^	0.882	0.429	0.002 *	0.001 *
Root length (mm)						
Male	14.55 ± 1.14	14.32 ± 1.39	14.47 ± 1.53	14.16 ± 0.88	14.18 ± 1.10	13.76 ± 0.92
Female	13.83 ± 1.65	13.86 ± 1.65	14.56 ± 1.87	14.67 ± 1.56	13.87 ± 1.30	13.73 ± 1.21
*p* value	0.151 ^†^	0.853 ^†^	0.897	0.335 ^†^	0.586	0.950 ^†^
Root–crown ratio						
Male	1.65 ± 0.22	1.61 ± 0.21	1.60 ± 0.16	1.55 ± 0.11	1.56 ± 0.14	1.47 ± 0.07
Female	1.61 ± 0.22	1.60 ± 0.22	1.62 ± 0.26	1.64 ± 0.21	1.63 ± 0.15	1.59 ± 0.14
*p* value	0.528 ^†^	0.902 ^†^	0.762	0.145	0.276	0.002 *

Data are presented as the mean ± standard deviation. Right and left measurements are shown separately. Normality was assessed using the Shapiro–Wilk test. Comparisons between males and females were performed using the independent *t*-test for normally distributed data (^†^) or the Mann–Whitney U test for non-normally distributed data. *: *p* < 0.05.

## Data Availability

The data used to support the findings of this study are included in the article.

## Abbreviations

The following abbreviations are used in this manuscript:

ANB	A point–Nasion–B point angle
ALARA	As low as reasonably achievable
CEJ	Cemento-enamel junction
CBCT	Cone-beam computed tomography
ICC	Intraclass correlation coefficient
OIEARR	Orthodontically induced external apical root resorption
PFA	Prefabricated functional appliance
R/C ratio	Root-to-crown ratio
SD	Standard deviation
